# Regulation of DNA Replication in Early Embryonic Cleavages

**DOI:** 10.3390/genes8010042

**Published:** 2017-01-19

**Authors:** Chames Kermi, Elena Lo Furno, Domenico Maiorano

**Affiliations:** Genome Surveillance and Stability Laboratory, Institute of Human Genetics, UMR9002-CNRS-UM, 141 rue de la Cardonille, Montpellier 34396, France; chames.kermi@igh.cnrs.fr (C.K.); elena.lo-furno@igh.cnrs.fr (E.L.F.)

**Keywords:** development, S-phase, cell cycle, *Xenopus*, *Drosophila*, translesion synthesis, checkpoint

## Abstract

Early embryonic cleavages are characterized by short and highly synchronous cell cycles made of alternating S- and M-phases with virtually absent gap phases. In this contracted cell cycle, the duration of DNA synthesis can be extraordinarily short. Depending on the organism, the whole genome of an embryo is replicated at a speed that is between 20 to 60 times faster than that of a somatic cell. Because transcription in the early embryo is repressed, DNA synthesis relies on a large stockpile of maternally supplied proteins stored in the egg representing most, if not all, cellular genes. In addition, in early embryonic cell cycles, both replication and DNA damage checkpoints are inefficient. In this article, we will review current knowledge on how DNA synthesis is regulated in early embryos and discuss possible consequences of replicating chromosomes with little or no quality control.

## 1. Introduction

The early embryonic cell cycles of most metazoans are usually contracted compared to those of somatic cells [1]. In the majority of animals, embryonic cell divisions are very rapid and highly synchronous (with some exceptions [2]) including a replication phase (S-phase) and a division phase (M-phase), with short or absent intermediate G1- and G2- (gap) phases [3]. These amazingly fast embryonic cell cycles, typical of animals with external development, can be explained as an adaptation to ensure the subsistence of laid eggs in the hostile external environment and the need to proceed to the hatching stage as quickly as possible. Mammalian embryonic cell cycles are longer, and, in this respect, they represent an exception to those of many other species. Probably the most astonishing feature of DNA replication in the early embryo is its speed. During the early cleavages of *Xenopus* embryos, DNA replication occurs in less than 30 min, which is about 20 times faster than in somatic cells [4]. If one may think that replicating the *Xenopus* genome in such a short time is a fast process, then it is even more astonishing to find out that the *Drosophila* genome is replicated in less than 4 min [5]. These observations raise the following questions: What makes DNA synthesis so fast in these embryos? Most importantly, what are the consequences of replicating the genome at such a high rate? These are two main points that we shall address in this review.

## 2. Onset of S-Phase in the Fertilized Egg

DNA synthesis in the laid egg is activated upon fertilization. After fertilization, the first mitosis is relatively slow in comparison to the following cell cycles. This extra time is necessary to complete the second meiotic division so to ensure decondensation of sperm chromatin and fusion of the male and female pronuclei to produce a diploid genome [2]. Nuclear fusion occurs in interphase before the first mitosis in sea urchin, *Caenorhabditis elegans*, and *Xenopus laevis* [6,7,8], whereas, in mammals, the nuclear envelope breaks down after the two pronuclei undergo DNA replication independently, and chromosomes then associate during the first mitosis [9,10,11,12].

Initiation of DNA replication in early embryos has been best studied and characterized in the clawed frog *Xenopus laevis*, mainly thanks to the development of *cell-free* extracts capable of recapitulating all the sequential steps of DNA synthesis leading to the formation of functional replication forks ([13] for review). The exceptional performance of these extracts relies on a very high abundance of most cellular proteins stored in the unfertilized egg. *Xenopus* egg extracts are also naturally synchronized in very early S-phase, so that in this system the dynamics of assembly of replication complexes can be analyzed in great detail and in a short time window.

**Figure 1:**
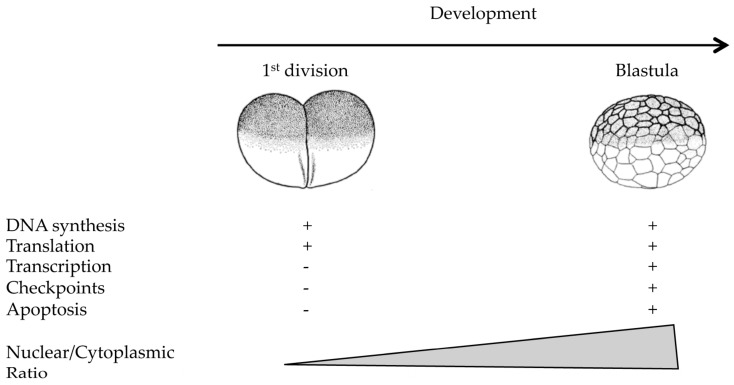
Reactivation of cellular processes during the early development of *Xenopus laevis*. Apart from DNA synthesis and translation, several cellular processes are inactive during the early stages of development. These processes are restored close to the time when zygotic transcription is activated, the midblastula transition in fast cleaving embryos such as *Drosophila* and *Xenopus*.

In *Xenopus* eggs, transcription is repressed and therefore S-phase depends upon a large stockpile of maternally-supplied proteins [14,15]. For instance, synthesis of histones is not required [16,17], as opposed to somatic cells where it is tightly coupled to S-phase onset. Transcription in the embryo is resumed after a series of 13 embryonic cleavages, close to the onset of the Mid Blastula Transition (MBT, Figure 1). During early mammalian development, transcription is also repressed, however only for the first zygotic cleavage in mouse, and up to the 4–8 cell stage in human [18,19].

### 2.1. Developmental Regulation of DNA Replication Origin Usage

**Figure 2:**
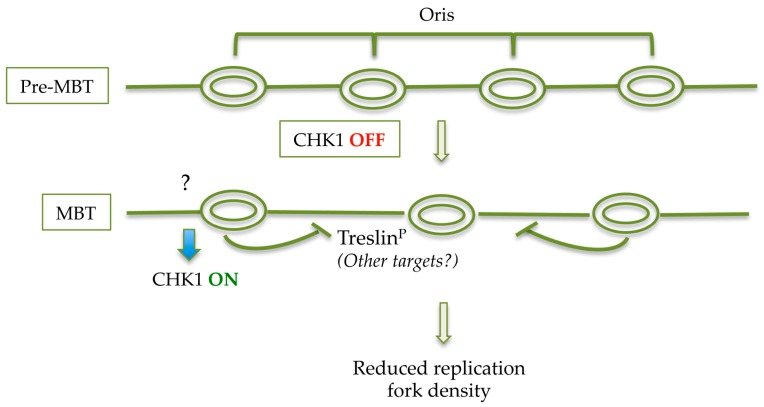
Speculative model of decreased origin density at MBT. Developmental activation of CHK1 at MBT stimulated by as yet unclear cues (question mark), induces local phosphorylation of Treslin (and probably of other targets that remain to be identified) which suppresses initiation of DNA synthesis within a replication cluster thus leading to a reduced replication origins density.

DNA replication initiates at multiple sites distributed along the chromosome, the DNA replication origins. These are the sites where replication complexes are assembled and DNA replication begins ([20], for extensive review). Work in *Xenopus* and *Drosophila* has unveiled one peculiar feature of DNA replication origins in embryos that contributes to the fast replication rate. In the early embryos of these organisms, replication origins are more abundant than in somatic cells, or at later stages of development [21,22]. Typically, replication origins in a 2-cell stage *Xenopus* embryo are regularly spaced every 10–15 kb [21,23,24,25,26], while in somatic cells this distance is increased about 10-fold [27]. This particular organization of the replicon results in an increased number of active replication origins and thus contributes to the accelerated rate of S-phase (Figure 2 and Table 1). Close to the MBT, the density of replication origins declines, initiation of DNA replication becomes restricted to specific sites and correlates with cell cycle lengthening [21,22]. Further, during very early embryogenesis, the feedback control that slows down the cell cycle if DNA replication has not been completed (intra S-phase checkpoint, see below) is not very efficient (Figure 1 and [28]). Full checkpoint activation is observed close to the MBT [29,30]. Previous observations have shown that checkpoint activation depends upon the Nuclear to Cytoplasmic ratio (N/C) which increases during development due to reduction of the cytoplasm volume in the cleaving embryos [15]. This regulation has two main consequences. The first is that there is no temporal regulation of origins firing orchestrated by the replication checkpoint. Indeed, analysis of DNA replication dynamics in nuclei assembled in *Xenopus* egg extracts at low N/C ratio, typical of early embryogenesis, has shown that clusters of active replication origins are more abundant and fire more synchronously compared to high N/C ratio, typical of post-MBT embryos. In addition, the speed of replication forks appears to be 3-fold faster (1 versus 3 kb/min [31]). Inhibition of the checkpoint by caffeine at high N/C ratio increased the density of origins firing, however it did not alter replication fork speed. Hence, an inefficient replication checkpoint contributes to the increased density of replication origins. The molecular determinants responsible for increased fork speed at low N/C ratio are not known. The second consequence of an absent checkpoint is that embryos have actually no means to arrest S-phase if chromosomes have not been completely replicated. It is therefore currently unclear whether the entire genome is completely and faithfully replicated in such a short cell cycle, which appears not to be the case in mouse ([32], see Section 6).

A study in *Xenopus laevis* has proposed that four DNA replication factors, Cut5/TopBP1, RecQL4/SLD2, Treslin/SLD3, and the DBF4 ortholog DRF1, become limiting after MBT onset and were proposed to be important to increase the replicon size [33,34]. However, the increase in replicon size observed at the MBT is rather modest (see Section 5.1.1), while dilution of essential replication factors is expected to produce a much greater effect on the inter origin distance. Hence, by itself, this hypothesis is not be sufficient to explain the expanded S-phase length, suggesting that other mechanisms may be implicated.

### 2.2. Assembly of Replication Forks in Early Embryos

The processes leading to formation of functional replication forks in the early embryo is similar to that observed in somatic cells. Remarkably, DNA replication is an evolutionary conserved process and studies using *Xenopus* egg extracts have been crucial in elucidating the mechanism of DNA replication in somatic mammalian cells [13]. Similar to somatic cells and unicellular organisms, formation of replication forks requires a sequential assembly of distinct multiprotein complexes at replication origins. These include pre-replication (pre-RC), pre-initiation (pre-IC), initiation (IC) and elongation (EC) complexes ([36], for review). However, some differences with somatic cells have been reported. While in somatic cells (and yeast) recruitment of the ssDNA binding protein RPA to replication forks depends upon S-CDK activity, required for origin unwinding, in *Xenopus* significant binding of RPA to chromatin occurs in a S-CDK-independent manner [37]. This is also the case for the essential replication factor Cut5/TopBP1 whose S-CDK-independent binding to chromatin is sufficient to allow normal DNA synthesis [38]. Virtually all known DNA replication proteins are found in large excess in the *Xenopus* egg. For instance, the ORC2 subunit of the Origin Recognition Complex (ORC), is present at over 94% excess in the egg cytoplasm compared to somatic cells [26]. In mouse embryonic stem cells (ESCs), very recent data show the presence of about two-fold more MCM2–7 helicase proteins chromatin-bound compared to differentiated neural progenitors, although the size of the replicon is comparable to that of somatic cells [39]. This suggests that the slight excess of MCM2–7 does not result in an increased number of active origins. The authors also reported that upon treatment with the DNA replication inhibitor hydroxyurea the size of the replicons of ESCs is slightly shorter than that of differentiated cells, suggesting activation of more dormant origins. However, it remains unclear whether this difference is due to the different cell cycle distribution of these cells types. A recent paper suggests that chromosome decondensation on metaphase exit in early *C. elegans* embryos depends on initiation of DNA replication, suggesting that the assembly of pre-RC components also facilitates chromatin remodeling [40], in line with a previous report in *Xenopus* [41].

Embryonic isoforms of four replication proteins have been reported. These are MCM3, MCM6, CDC6 and DBF4. In *Xenopus* and *Zebrafish*, maternal MCM3 lacks a nuclear localization signal compared to somatic MCM3 [42]. Interestingly, overexpression of maternal MCM3 interferes with DNA replication and causes developmental defects, while overexpression of somatic MCM3 (or maternal MCM3 containing the C-terminal of somatic MCM3 that lacks the NLS) has very little effect. A zygotic form of MCM6 (*zmcm6*) is expressed only after gastrulation but its function is unknown [43]. Two isoforms of CDC6, A and B, coded by two distinct genes, have been identified in the *Xenopus* egg. The B isoform appears at the gastrulation stage replacing the maternal A isoform [44]. The difference between these two isoforms resides mainly in the amino-terminal part of the protein that contains both regulatory signals for its phosphorylation by S-CDKs and a destruction box that targets CDC6 for degradation upon S-phase entry. The zygotic form of CDC6 contains a KEN box that targets it for proteasomal degradation, while in the maternal form of CDC6 this sequence is mutated and may explain its stability during early development.

In addition to DBF4, a second CDC7 activator called DRF1 is essential during very early *Xenopus* development, forming the active kinase also known as DDK. DRF1 is required for DNA synthesis in pre-MBT embryos, while after gastrulation, DRF1 levels drop sharply and CDC7-DBF4 becomes the most abundant kinase [33,45].

### 2.3. Once-Per-Cell Cycle Regulation of DNA Replication in Early Embryos

DNA replication must be limited to only one round per cell cycle in order to maintain a stable ploidy. Despite the high concentration of replication proteins, DNA replication still occurs only once per cell cycle in the early embryo [46] as in somatic cells, meaning that some regulatory mechanisms must exist to limit the activity of abundant proteins that may stimulate DNA synthesis before cell division. This is the case, for instance, for the essential pre-RC component Cdt1. Cdt1 appears to be limiting for activation of DNA synthesis and for the once-per-cell cycle regulation of chromosome replication during early *Xenopus* development. Even a small increase in the amount of Cdt1 in the egg results in aberrant re-initiation of DNA synthesis [47,48]. Cdt1 activity is finely regulated by at least two mechanisms. First, proteolytic removal of chromatin-bound Cdt1 after initiation of DNA synthesis [49,50], which depends upon interaction with PCNA and the DDB1 ubiquitin ligase that targets Cdt1 for degradation [51]. Second, Cdt1 activity is also regulated by interaction with the Geminin protein, which acts as an inhibitor of Cdt1 [52,53,54]. Geminin is also regulated by proteolytic degradation at mitotic exit by the activity of the Anaphase Promoting Complex (APC^CDC20^; [55]). In somatic cells, complete Geminin degradation gives to Cdt1 a short window of opportunity to promote pre-RCs formation and therefore initiate DNA synthesis. Geminin is stabilized in S- and G2/M-phases, when APC^CDC20^ activity is very low, thus imposing a block to re-initiation of DNA synthesis within the same cell cycle [52,55]. During early *Xenopus* development maternal Geminin is not completely degraded at each mitotic exit [56,57], and yet cytoplasmic Cdt1, whose levels remain unchanged [48,57], can still promote the initiation of DNA synthesis. Of note, in the early embryonic cleavages of *Xenopus*, proteolytic degradation of Cdt1 is inefficient, making Geminin a main regulator of Cdt1 activity by regulated change in the stoichiometry of the Cdt1:Geminin complex, while regulated proteolysis is resumed mainly close to the MBT [58,59].

## 3. Positive and Negative Regulation of Replication Initiation by S-CDKs and CHK1

The activity of S-CDKs is required for activation of S-phase during early *Xenopus* development [60,61]. S-CDKs targets include components of the pre-IC, such as SLD2/RecQL4, SLD3/Treslin/Ticrr and DUE-B. However, several differences exist in the regulation of cyclins activity in embryos compared to somatic cells. First, in the early embryonic cycles, S-phase cyclins are present in large excess and do not fluctuate as opposed to somatic cells. In *Xenopus*, only mitotic Cyclin B1 and B2 oscillate and their degradation leads to mitotic exit [62,63]. Second, only two CDKs, CDK1 and 2, and three cyclins (A, B and E) are present in the early embryo. Two forms of Cyclin A, A1 and A2, are found during very early embryogenesis of which Cyclin A1 is almost exclusively associated with CDK1. At the MBT both maternal Cyclins A disappear and are replaced by zygotic Cyclin A2 that associates with CDK2 and is involved in S-phase regulation [64]. The identification of zygotic-specific Cyclin E, Cyclin E2, has also been reported in *Xenopus* [65]. While maternal Cyclin E1 is constitutively present during early embryogenesis, Cyclin E2 appears at MBT and is required for gastrulation. These data also demonstrate an essential role for Cyclin E in the development of *Xenopus*, while Cyclin E seems to be dispensable for viability in mice [66,67]. The dependency of CDK activity upon DNA synthesis was demonstrated using *in vitro* egg extracts by removal of CDKs by p13^suc1^-coupled Sepharose beads [60,61,68,69]. It was later shown that both Cyclin A and E, but not Cyclin B, could provide S-phase-promoting (SPF) activity [70]. Intriguingly, in yeast it was shown that Cyclin B can also provide SPF activity [71]. This apparent contrast was later resolved by showing that also in *Xenopus* Cyclin B can provide SPF activity if its nuclear translocation is forced [72]. This experiment elegantly demonstrated that Cyclin B is biochemically functional in providing SPF activity. The difference between yeast and multicellular organisms is probably due to the absence of nuclear membrane breakdown in yeast at mitosis.

**Figure 3:**
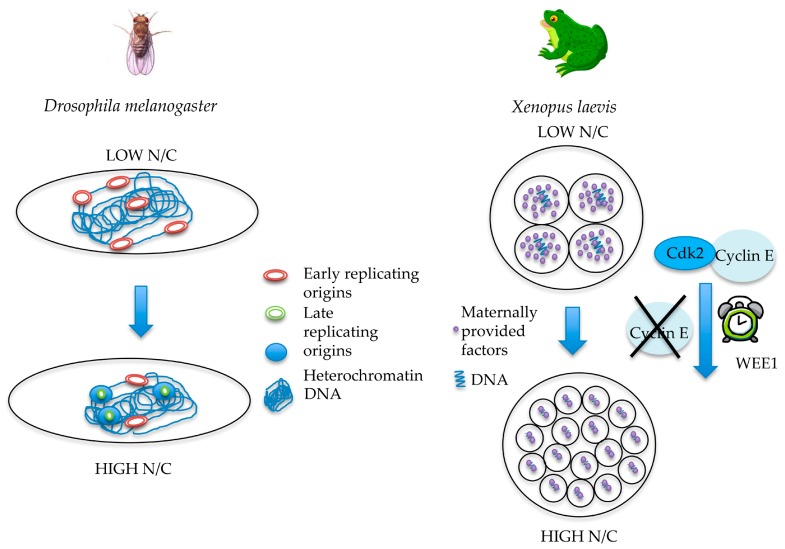
Schematic representation of mechanisms leading to S-phase lengthening at the MBT in *Drosophila* and *Xenopus*. In *Drosophila,* the establishment of heterochromatin, concomitant to the increase in the N/C ratio, contributes to S-phase lengthening after MBT. In *Xenopus,* titration of maternally inherited factors by the increased N/C ratio and degradation of Cyclin E participate in increasing S-phase length at the MBT. The WEE1 gene is implicated in regulating the timer for MBT onset by acting on the stability of the Cyclin E/CDK2 complex.

Interestingly, Treslin has been very recently reported to be also a substrate of CHK1. Mutation of the Treslin CHK1 binding site stimulated initiation of DNA replication by increasing both the loading of CDC45 onto chromatin and the number of active clusters of replication origins, but did not have an effect on replication fork speed [18,73]. These latter findings put forward Treslin as a target responsible for the checkpoint-mediated reduced fork density observed at the MBT (Figure 3).

## 4. DNA Replication-Dependent Inheritance of Epigenetic Marks: Methylation Program

During early embryogenesis, a wave of epigenetic reprogramming is established allowing the cells of the early embryo to remain pluripotent and as such prevent premature differentiation. This occurs primarily by downregulation of the DNA methyltransferases that passively promote global demethylation of maternally inherited DNA over several cycles of DNA replication [11,74,75]. Hence, during the early embryonic cleavages, epigenetic marks, such as modification of histone tails by methylation, are not established, nor maintained during DNA replication.

## 5. Mechanisms Leading to S-Phase Lengthening at the Mid Blastula Transition

### 5.1. Similarities and Differences between Different Organisms

As mentioned in the previous paragraphs, the extremely fast S-phases that characterize the first dozen of early embryonic cycles in fast cleaving embryos experience a severe slow down when the transcription of the zygotic genome is activated for the first time (Zygotic Genome Activation, ZGA). In addition to full activation of the replication checkpoint, additional hypotheses have been put forward to explain both cell cycle slow down and reduced replication forks speed. These include, exhaustion of limiting replication factors and/or chromatin components, dilution of key cell cycle regulators and ZGA. However, a clear picture has not emerged and the mechanism(s) implicated may probably be divergent in different organisms. Different developmental strategies employed by different organisms as well as evolutionary features may account for this divergence.

#### 5.1.1. *Drosophila melanogaster*

During the earliest cycles of *Drosophila melanogaster*, embryos form a syncytium in which nuclei are not surrounded by a cell membrane [76]. In this context, DNA replication occurs within nuclei that are embedded into the cytoplasm of the syncytium. It is only following MBT that S-phase slows down. This maternal-to-zygotic transition (MZT) is more like a succession of progressive events rather than a sudden single change [3]. Two mechanisms have been put forward to explain S-phase lengthening after MBT in *Drosophila*. The first is the increase in inter-origins distance, from 8 kb in the preblastoderm embryo [5], to about 10 kb at cycle 14 [77]. Thus, it would take longer to replicate between origins after MBT. However, by itself, this change could not explain the enormous increase (~15 fold) in the length of S-phase between the first cell cycles and cycle 14. Second, the MBT timing in *Drosophila* (as in *Xenopus*) is dependent on the N/C ratio, and not on zygotic transcription as it was shown by performing injection of α-amanitin (an RNA polymerase II inhibitor) in the embryos to inhibit RNA synthesis [15].

Replication timing of different genomic sequences may play an important role in S-phase lengthening during *Drosophila* embryogenesis, consistent with the observed reduced synchrony of clusters of replication origins firing also observed in *Xenopus* [31]. In somatic cells, as in post-MBT embryos, specific DNA regions replicate at different time points during S-phase. Euchromatin-embedded genes are the first to replicate upon the onset of S-phase, whereas heterochromatin sequences are replicated at a later time [78]. In contrast, both euchromatin and heterochromatin replicate at the same time in the preblastoderm embryos. While the embryo is developing, satellite DNA sequences progressively shift from being early replicating to late replicating, and then after MBT clusters of satellite sequences dramatically turn to late-replicating sequences [78,79,80]. This shift correlates with the establishment of replication-dependent methylation in late embryos. In the pre-MBT cycles, the shift is gradual and subtle, and replication of euchromatin and satellite sequences still largely overlaps. The change is dramatic after MBT, when different clusters of satellite sequences replicate in late S-phase [78,79,80]. For instance, in *Drosophila* certain late sequences start replicating between 15 and 30 min after the beginning of S-phase in cycle 14, a period of time longer than the entire S-phase of cycle 13 [78].

#### 5.1.2. *Xenopus laevis*

*Unlike Drosophila*, *Xenopus* embryos undergo complete cellularization since the first embryonic cleavages. The first 12 cell cycles are fast and synchronous, alternating between DNA replication and cell division at 30 min intervals until the MBT [15], when cell cycles progressively slow down (50, 99, and 253 min for cycles 13, 14, and 15, respectively [64]). Cycle 15 corresponds to the onset of gastrulation. The MBT was defined in *Xenopus* as the initial slowing of the cell cycle concomitant to ZGA onset and cellular movements [29]. Nevertheless, these three events have been subsequently shown to be temporally uncoupled in both *Xenopus* [81] and *Drosophila* [82]. In addition, another dramatic change in the cell cycle, related to Cyclin A regulation, occurs in the *Xenopus* embryo after MBT and just prior to gastrulation, called the Early Gastrula Transition (EGT, [64]). In comparison to *Drosophila*, similar changes close to MBT are observed in *Xenopus* at cycle 10, called pre-MBT slowing, and it would be more appropriate to compare the *Drosophila* cycle 14 embryos with the EGT changes in *Xenopus* [3]. Exhaustion of the replication factors TopBP1, Treslin, DRF1/DBF4 and RecQL4 has been proposed to explain S-phase lengthening leading to activation of the checkpoint in *Xenopus*. Dilution of these factors correlates with slowing down of the cell cycle, and zygotic replication initiation. Overexpression of these factors induces additional short pre-MBT-like cycles without accelerating the pre-existing pre-MBT cycles and delays the onset of transcription [34]. The specificity of these factors in inducing extra cycles of replication after the MBT remains to be tested.

#### 5.1.3. *Zebrafish*

In *Zebrafish*, the embryo initially goes through 9 rapid and synchronous cell cycles. The cell cycle starts slowing down slightly during the 10^th^ and 11^th^ division, before undergoing massive cell cycle changes, zygotic transcription and initiating cell movements. Cell cycle asynchrony appears first in cycle 11 [83]. As in *Drosophila* and *Xenopus*, in *Zebrafish* MBT onset also depends upon the N/C ratio as suggested by partial enucleation experiments [83]. In addition, in *Zebrafish* embryos, injection of α-amanitin did not delay MBT onset, thus showing that this transition is independent from ZGA [84,85]. The G1-phase of the cell cycle is introduced for the first time at MBT in a transcription-dependent manner, suggesting that the cell cycle slowing at the MBT does not depend upon the appearance of this gap phase [72].

#### 5.1.4. Mammals

The length of S-phase in mammalian early embryonic cleavages is variable from one cell cycle to another, and significant differences have been reported between mouse and human embryos. Nevertheless, transcriptional quiescence in early embryonic development is an evolutionarily conserved phenomenon. During mouse embryonic development, ZGA starts at the two-cell stage [86], so that the length of S-phase between cycles 1 and 2 can be remarkably different (Table 1). In human embryos ZGA occurs at a stage between 8 and 16 cells [19], hence the length of S-phase increases at later stages than in mouse. These differences may also explain the observed divergence in both the pluripotency regulatory network [87] and the efficiency of different checkpoints between mouse and human embryos [88,89].

### 5.2. The Role of CDKs

S-phase lengthening at MBT may also be influenced by developmental changes in S-CDK activity by targeting components of the pre-IC complex, such as RecQL4/SLD2 and Treslin/Ticrr. Cyclin E overexpression is sufficient to induce unscheduled entry into S-phase in mammalian somatic cells [73]. Hence, because in early embryos Cyclin E is overexpressed, it is possible that its abundance has also a positive effect on the speed of S-phase. Cyclin E/CDK2 accumulates during the first embryonic mitotic cycle and remains stable until MBT in *Xenopus* [63,90]. Despite the fact that Cyclin E levels remain stable, Cyclin E/CDK2 activity changes, with two peaks, in S-phase and mitosis [91]. However, it has been shown that in *Xenopus* extracts Cyclin A/CDC2 is more involved in DNA replication than Cyclin E/CDK2 [70,92]. Cyclin E1 is degraded during the MBT [63,90] and this degradation is independent from the N/C ratio, cell cycle regulation, zygotic transcription, or *de novo* protein synthesis [93]. Using the *Xenopus* CDK inhibitor Xic1 [94], Hartley and colleagues suggested that Cyclin E/CDK2 regulation in early embryogenesis is linked to “an autonomous maternal timer” driving the early embryonic cleavages until the MBT [95]. A more recent study has suggested that the Wee1 kinase disrupts Cyclin E/CDK2 activity near MBT [96].

### 5.3. The Role of the Replication Checkpoint

Activation of the replication checkpoint affects the progression of S-phase. Checkpoint signals are triggered by a DNA replication block or DNA damage to prevent origin firing through an inhibitory pathway that depends upon the PI3K kinases ATM, ATR and DNA-PK [31,97]. Normal progression of DNA synthesis is mainly monitored by ATR. In situations where the enzymatic activity of replicative DNA polymerases becomes uncoupled from that of the CMG helicase (replication fork uncoupling) formation of excess ssDNA occurs, which constitutes an essential substrate required to activate ATR ([36] for review). Small replication intermediates are then generated on the lagging strand by DNA polymerase α and δ, and stabilized by at least one translesion synthesis DNA polymerase [98,99,100]. These intermediates are then recognized by the RFC^Rad17^ clamp loader that allows loading of the essential checkpoint clamp 9-1-1, leading to full ATR activation through its tethering to TopBP1 and ATRIP proteins. Activation of ATR leads to phosphorylation of many substrates, amongst these the CHK1 kinase. This latter regulates the stability of the CDC25A protein phosphatase that in turn regulates the phosphorylation state of CDK2. A DNA replication block or slow down activates ATR, ultimately leading to degradation of CDC25A and inhibition of Cyclin E/CDK2 activity, which inhibits origin firing [97,101].

**Figure 4:**
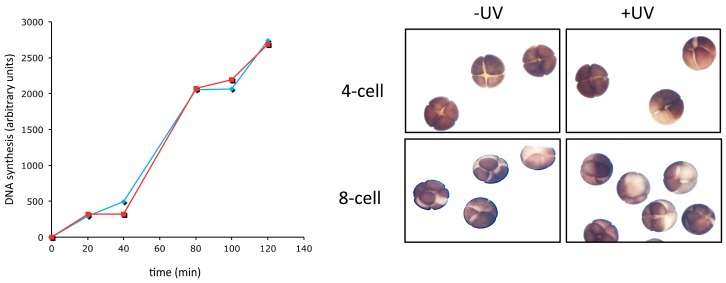
S- and M-phases of early embryos are insensitive to DNA damage. DNA synthesis (**left**); and images of *Xenopus laevis* embryos fertilized in vitro (**right**), cleaving in the absence (blue line and −UV) or presence (red line and +UV) of moderate doses of UV-C irradiation (300 J/m^2^). Exposure to higher UV doses results in a cell cycle block due to non-specific cross-link of proteins to chromatin and failure to decondense chromosomes.

Both the replication and the DNA damage checkpoint are inefficient in early embryos of fast cleaving organisms [4,30,81,102,103], as well as in mammalian embryonic stem cells [88,89]. For instance, the replicative DNA polymerases inhibitor aphidicolin does not slow down the early embryonic cleavages in both *Drosophila* [30] and *Xenopus* [55]. Consistent with this original observation, DNA synthesis in *Xenopus* egg extracts at low N/C ratio is insensitive to moderate doses of UV irradiation and does not slow down the cell cycle ([104] and Figure 4). Similarly, *C. elegans* embryos are not sensitive to high doses of both the alkylating agent MMS and UV light [105,106]. These checkpoints become fully operational close to the MBT [107,108]. Their activation occurs in two phases: a pre-MBT gradual one, and an abrupt slowing at MBT. The first phase is linked to the gradual activation of the CHK1 pathway. Prior to the MBT, DNA replication activates the replication checkpoint progressively giving the impression of a gradual lengthening. Consistent with this possibility, *grapes* (CHK1) *Drosophila* mutant embryos never hatch and undergo mitotic catastrophe in mitosis 13 due to a premature entry in M-phase with incompletely replicated chromosomes [109,110]. These embryos fail to delay mitosis until completion of replication because, in the absence of Grapes, CDK1 is not phosphorylated and thus inhibited [109,111]. Furthermore, *grapes*-mutated embryos fail to prolong pre-MBT cycles as in normal embryos [111] suggesting a major role of Grapes-driven inhibitory phosphorylation in pre-MBT interphase lengthening.

The abrupt cell cycle slow down at MBT correlates with CDC25-dependent CDK1 inactivation and, as a consequence, introduction of a G2-phase and DNA replication slow down [112,113]. Consistent with this observation, in *Drosophila*, the two CDC25 orthologs String and Twine are expressed at high levels during the pre-MBT cycles [114,115,116]. Twine levels remain high until early MBT, when it is rapidly destroyed, whereas String levels progressively decline until disappearing prior MBT [114]. Therefore, Twine protein appears to be responsible for CDK1 inhibition that lengthens S-phase, and adds G2-phase at MBT in *Drosophila melanogaster*.

The molecular determinants that induce developmental CHK1 activation at the MBT remain unclear, although some candidates emerge. The model of replication factor exhaustion is unlikely to be a major contributor to this regulation since observations in *Drosophila* and *Xenopus* suggest that origin spacing increases very little just after MBT [77]. Further, in *Zebrafish*, no connection between the N/C ratio and S-phase lengthening, or between the N/C ratio and CDC25/CDK1 destabilization is clearly established. In contrast, it has been shown that upregulating CDC25A activity or expressing an inhibitory phosphorylation-resistant *cdk1* mutant causes continued rapid divisions [85], pointing out to a role of CDC25A and CDK1 inhibition in cell cycle lengthening and asynchrony between the cycles 9 and 12. Of note, zygotic transcription initiation is not required for cell cycle lengthening.

**Figure 5:**
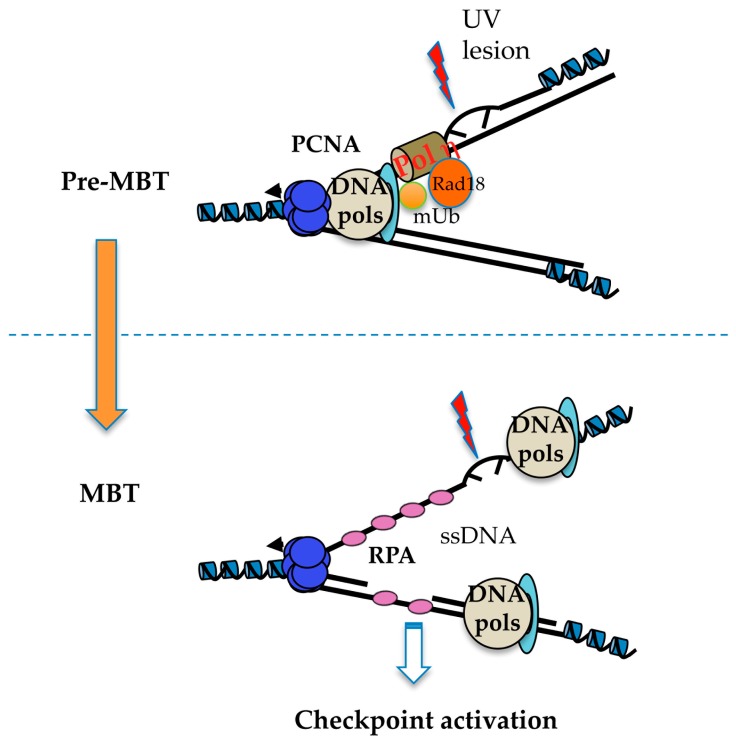
A DNA damage-tolerant replisome in early embryos? Speculative representation of replisome structure in pre- and post-MBT embryos. In pre-MBT embryos, constitutive PCNA^mUb^, driven by high RAD18 expression, allows recruitment of TLS Pol η to replication forks thus limiting replication fork uncoupling in front of DNA damage (UV-C lesions in this example). In this situation, formation of ssDNA, which is a prerequisite for checkpoint activation, is strongly reduced. In post-MBT embryos that contain reduced RAD18 levels, PCNA^mUb^ requires ssDNA formation, thus leading to checkpoint activation.

The molecular mechanisms responsible for checkpoint inhibition in early embryos are poorly understood. Using *in vitro* and *in vivo* experiments in *Xenopus* [103,107,117], checkpoint activation has been shown to be independent of transcription or translation, and to pertain to the N/C ratio. This is due to the exponential increase of the amount of DNA that doubles every cell cycle without significant cell growth, suggesting titration of maternal limiting factors of unknown identity. Addition of a threshold amount of undamaged DNA allows a DNA damage checkpoint response to be activated confirming the titration model. Genetic studies in the worm *C. elegans* have involved RAD-2, GEI-17 sumo E3 ligase, and the translesion DNA polymerase POLH-1 (TLS Polη) specialized in the replication of damaged DNA [106,118,119]. Some of us have recently shown that the RAD18 ubiquitin ligase, a master regulator of the DNA damage tolerance pathway that involves translesion DNA synthesis, and not TLS Polη, is limiting for activation of the checkpoint sensing the presence of DNA damage in the *Xenopus* embryo. High levels of maternally deposited RAD18 present in the embryo induce both constitutive PCNA^mUb^ and consequent recruitment of TLS Polη onto chromatin thus making replication forks DNA damage-tolerant. The mechanism involves inhibition of replication fork uncoupling that, by inhibiting formation of excess ssDNA, does not allow full checkpoint activation [104]. Constitutive PCNA^mUb^ can also be observed in *Drosophila* pre-MBT embryos (Lo Furno, Busseau and Maiorano, in preparation). RAD18 abundance is developmentally-regulated. It decreases at stage 6, well before MBT, and may depend on proteolysis. Hence, these observations suggest that replication forks in early embryos of *Xenopus*, *Drosophila*, and *C. elegans*, may be DNA damage-tolerant (Figure 5). This regulation may also contribute to increased fork speed in pre-MBT embryos. Importantly, does not appear RAD18 to be involved in the developmental activation of CHK1 observed at MBT, suggesting that DNA damage-tolerant replication is not responsible for the reduced origin density observed before MBT. We propose that this latter process may occur in two steps. The first step involves checkpoint derepression induced by decreased RAD18 levels close to MBT, while the second step implicates stalling of replication forks that induces CHK1 phosphorylation by ATR activation. The nature of replication fork stalling at MBT remains to be identified.

### 5.4. The Role of Zygotic Transcription Activation

Activation of zygotic transcription close to MBT could contribute to S-phase lengthening by interfering with the assembly of DNA replication origins and therefore reducing the inter-origins spacing. Another possibility is that activation of zygotic transcription triggers by itself a checkpoint signal that slow down S-phase. Recent work in *C. elegans* [120] and *Drosophila* [121] has suggested that activation of transcription triggers activation of the replication checkpoint. Blyte and Wieschaus [121] proposed that stalling of replication forks at genes poised by RNA polymerase II would trigger a checkpoint response leading to activation of CHK1, thus resulting in S-phase lengthening by downregulation of CDC25A activity. The onset of zygotic transcription seems to be a gradual process in which genes initiate expression at different times. Based on a high-throughput study comparing the expression of many genes in wild-type versus haploid embryos, genes were divided in two categories: genes whose transcription is dependent on N/C ratio and time-dependent genes. Some genes were expressed one cycle later in haploid embryos, whereas others kept normal transcription timing independently from DNA amount [122]. Accordingly, Twine (CDC25) degradation could be dependent on expression of N/C dependent genes consistent with the notion that cell cycle slowing requires activation of transcription. This model is supported by the observation that haploid embryos show delayed Twine degradation [115].

How the N/C ratio could control transcription and induce cell cycle remodeling leading to MBT onset is still puzzling. Several models have been proposed to explain the onset of ZGA in early embryos. One model is titration, on the exponentially increasing DNA, of some maternal components stored in a limited amount in the embryo that serve as a sensor for N/C ratio and trigger transcription of N/C dependent transcripts thereby promoting zygotic genome activation and cell cycle remodeling. This possibility suggests the existence of transcription repressors in the early embryos that silence the genomic DNA and are subsequently titrated allowing ZGA. Previous [123,124,125] and more recent observations [126] suggest that these may be histones that out compete the binding of transcription factors to chromatin. Consistent with this model, increasing the DNA content of an embryo by inducing polyspermy, or by injecting large amount of DNA, is sufficient to induce an earlier onset of transcription [15]. Nevertheless, ectopic CDC25 expression in MBT *Drosophila* embryos is sufficient to introduce extra short cell cycles [113] arguing that the titration is not directly responsible for cell cycle remodeling.

Another model proposes that an autonomous molecular maternal timer is triggered just after fertilization and regulates the events preceding the MZT. This is confirmed by the fact that both Cyclin A and E1 degradation is independent from the N/C ratio and depends upon the time after fertilization [64,93,127]. Furthermore, work in *Drosophila* favors the “maternal timer” model rather than titration [122].

A third model links transcription silencing to the DNA replication machinery, and is supported by experiments showing premature zygotic transcription in *Xenopus* and *Drosophila* embryos blocked in interphase with cycloheximide [81,82]. In this model, it is proposed that the rapid DNA synthesis of early embryonic cleavages is responsible for abortive transcription, and that replication slows down close to MBT allowing completion of transcription.

Additional regulations must exist implicating other proteins. Among them are Zelda and Smaug. Zelda (Vielfaltig) is a zinc-finger DNA-binding protein, which binds specific sites on the genome and is highly enriched at genes that are expressed during the pre-MBT and the 14th cycle in *Drosophila* [128,129]. It is possible that Zelda serves as a binding platform for other transcription factors [130,131,132]. Increasing the number of Zelda binding domains induces premature transcription of the target gene. Conversely, removing Zelda binding sites near a gene delays onset of its transcription [133]. Certain *zelda*-mutated embryos show an extra pre-MBT rapid cell cycle, suggesting that one, or several genes involved in MBT timing, are regulated by Zelda [134,135].

Smaug has been also proposed as a timer of the MBT [136]. Smaug is an RNA-binding protein that promotes RNA destruction by shortening the poly(A^+^) tail through recruiting the CCR4/POP2/NOT deadenylase complex. *Smaug*-mutated embryos fail to efficiently activate the DNA replication checkpoint and do not show cell cycle slowing and MBT onset. Because the replication checkpoint plays an important role in regulating the embryo cell cycles, the role of Smaug could be indirect through the Grapes (CHK1) pathway. In addition, these embryos present a defect in the onset of zygotic transcription. However, the molecular basis of Smaug function in DNA replication checkpoint and transcription and its regulation by the N/C ratio are not well understood.

## 6. Consequences of Fast Replication and Absence of Checkpoint Activation on Early Embryos Genome Integrity

A fast replication mode, with little or virtually absent quality control (inefficient checkpoint) typical of early embryonic cell cycles, raises the question on how the embryos manage to preserve genome integrity during early development. In addition, the observation that embryos of some species also constitutively recruit TLS Pols into the replisome makes the situation worse since TLS is error-prone, which also implies that mutations may be generated during the early embryonic cleavages. The first question is whether early embryos manage to completely replicate their genome in a very short cell cycle. To date the best evidence stems from observations in mouse embryonic stem cells (ESCs). In these cells, the length of S-phase is similar to that of somatic cells, of about 8 h, however G1- and G2-phases are highly contracted [137]. Previous data have shown that ESCs accumulate a high level of DNA damage, visible as H2AX phosphorylation (γH2AX) and 53BP1 foci, higher than the damage generated in differentiated mouse embryonic fibroblast exposed to 1 Gy of γ irradiation [138,139]. More recent data have confirmed these observations and shown that the ATR kinase is responsible for high γH2AX levels, suggesting the presence of replication stress. This was also shown to be the case in the pre-implantation embryo [32,138]. More detailed molecular analysis showed that mouse ESCs accumulate multiple ssDNA gaps, each of about 0.5 kb in length, in 80% of replication forks analyzed. Assuming an inter origins distance of about 12 kb [27], and assuming that a bidirectional replication fork accumulates at least one ssDNA gap, this observation suggests that in mouse ESCs at least 10% of the genome is undereplicated. In addition, a high degree of reversed forks were also observed, as well as a great number of RPA and RAD51 nuclear foci [32]. A similar situation has been observed in human ESCs that have also been reported to have a highly unstable genome and an inefficient S-phase checkpoint [88,140]. At the molecular level, the consequences of genomic instability of early human embryos are formation of truncated chromosomes, often rescued by fusion of replicated sister chromatids resulting in dicentric isochromosomes, as well as formation of centromere-less chromosomal fragments. These abnormalities are strongly associated with DNA damage and poor developmental potential [141]. Other patterns are characterized by breakage-fusion-bridge products, with both terminal imbalances and terminal deletions, accompanied by inverted duplications [140]. Phenotypically, the consequences are a low fertility rate (only 30% of human conceptions result in a live birth) and spontaneous abortions. Induced pluripotent stem cells (iPSCs), generated by reprogramming of somatic cells, also show high levels of γH2AX and genomic instability ([142] for a review). Incidentally, the genomic instability of human ESCs and iPSCs raises important questions about the use of these cells in regenerative medicine. In fact, in addition to having an unstable genome, these cells generate teratoma when injected into mice. Altogether, these observations suggest that DNA replication in ESCs is incomplete, which raises the question of how these cells can cope with such a high level of DNA damage and produce viable embryos. One mechanism to preserve genome integrity upon exit from early embryogenesis is apoptosis. Not all ESCs differentiated *in vitro* are viable, but many of them are eliminated by apoptosis. In *Xenopus,* an apoptotic program is activated at the MBT onset that eliminates all cells having accumulated a high degree of DNA damage [143,144]. In *Drosophila,* damaged nuclei sink inside the blastoderm and thus become excluded from the developing embryos [145]. Hence, the toll to pay for replicating fast and in an inaccurate way is to accumulate DNA damage suggesting that replication in the early embryo may be inaccurate and may generate more errors than previously thought.

## 7. Conclusions

Early embryos modify the cell cycle as an adaption to the specialized features of early embryogenesis. This adaptation is related to the absence of transcription and the absence of differentiation programs that are activated later during embryogenesis. In rapid cleaving embryos a short inter origin distance, generated as a consequence of an inefficient replication checkpoint, and a fast replication fork speed contribute to the accelerated rate of S-phase. Although the molecular determinants responsible for increased replication fork speed remain to be identified, constitutive translesion synthesis is a possible candidate. In addition, DNA synthesis in the early embryo is DNA damage-tolerant and may be error-prone. In mammalian embryos, S-phase is longer, yet DNA accumulates damage and chromosomal abnormalities, probably due to cell cycle contraction and inefficient checkpoint response. These features suggest that DNA replication during early embryogenesis may not be completely faithful and raise important questions about the degree of mutation carry over in differentiated cells and its consequences. Does this represent an additional mechanism by which genetic variation is generated? Alternatively, is this the Achilles’ heel of evolution?

## Figures and Tables

**Table 1:** S-phase length during early animal embryonic development and compared to somatic cells. The length of S-phase in different organisms is indicated. Measurements in mammalian embryos are less precise due to the difficulty to obtain embryos and to the different experimental conditions employed. ZGA indicates activation of zygotic transcription. n.d.: not determined.

Organism	Cycle 1	Cycle 2	Cycle 3–4	Blastula/Blastocyst	Somatic Cell
*Drosophila* [3]	3.4 min	3.4 min	3.4 min	50 min (ZGA)	8 h
*Xenopus* [15]	20 min	20 min	20 min	210 min (ZGA)	8 h
Mouse [10]	4–7 h	1–5 h (ZGA)	n.d.	8 h	8 h
Human [35]	7–8 h	n.d.	(ZGA, n.d.)	8 h	8 h

## Abbreviations

The following abbreviations are used in this manuscript:

53BP	p53 binding protein
APC	anaphase-promoting complex
ATM	ataxia telangiectasia mutated
ATR	ataxia telangiectasia mutated- and Rad3-related
ATRIP	ATR-interacting protein
CCR4	C-C chemokine receptor type 4
CDC	cell cycle division
CDK	Cyclin Dependent Kinase
CDT	CDC10-dependent transcription
CHK	Checkpoint kinase
CMG	CDC45-MCM-GINS
DBF	dumbbell factor
DDB	DNA damage binding
DDK	DBF4-dependent kinase
DNA-PK	DNA-dependent protein kinase
DRF	dumbell-related factor
DUE	DNA unwinding element
Gy	Gray
kb	kilobase
MCM	mini chromosome maintenance
MMS	methyl methane sulphonate
mUb	monoubiquitination
NOT	Negative Regulator Of Transcription 1
ORC	origin recognition complex
PCNA	proliferating cell nuclear antigen
POP	posterior pharynx defect protein
RPA	replication protein A
RF-C	replication factor C
Rad	Radiation sensitive
ssDNA	single stranded DNA
Sld	synthetic lethal with Dpb11
Ticrr	TopBP1 interacting checkpoint and replication regulator
TopBP1	Topoisomerase II binding protein 1
9-1-1	Rad9-Rad1-Hus1
